# Unmet Needs in Mental Health: A Cross‐Sectional Study on Contact Coverage Gaps and Access to Care for Depression and Anxiety Among Adults in Kailali, Nepal

**DOI:** 10.1002/brb3.71551

**Published:** 2026-06-19

**Authors:** Gayatri Khanal, Y. Selvamani, Prabhat Sapkota

**Affiliations:** ^1^ School of Public Health, Faculty Medicine and Health Science SRM Institute of Science and Technology Kattankulathur Tamil Nadu India; ^2^ Nisarga Hospital and Research Centre Pvt. Ltd. Dhangadhi Nepal

**Keywords:** anxiety, barriers, contact coverage gap, depression, Kailali, mental health

## Abstract

**Background:**

There is a pronounced gap between demand and actual mental health service utilization, with earlier estimates suggesting that nearly 90% of affected individuals with depression or anxiety do not receive the care they need despite being the most prevalent and highest contributor for global burden. The present study aims to identify contact coverage gap and explore barriers while seeking care among those who screen positive for depression and anxiety.

**Methods:**

A community‐based cross‐sectional study was conducted among 1304 adults aged 20–60 years who had resided in Kailali, Nepal for at least 6 months. Multistage random technique was utilized to select the participants from three rural municipalities and three municipalities. Depression and anxiety were assessed by using widely recognized screening tools, namely, the Patient Health Questionnaire‐9 (PHQ‐9) and the Beck Anxiety Inventory (BAI). Barriers to care were measured using the Barriers to Access to Care Evaluation (BACE).

**Results:**

Out of 1304 study participants, around 15.2% were screened positive for at least one (depression and/or anxiety) illness. Screening positive for depression (13.3%) was higher than anxiety (7.5%). Only 28.3% of individuals who were positive for anxiety and/or depression contacted with service providers. Among those who sought care, most had visited traditional healers (98.2%). Major barriers to care were unaffordable cost (38.4%), self‐solving attitude (22.2%), preference for alternative types of care (21.7%), busy schedule with work (16.7%), speculating it will resolve itself (15.2%), and not being sure where to seek for mental health services (12.6%). Contact coverage was significantly associated with morbidity and lack of awareness on available services.

**Conclusion:**

There was a significant contact coverage gap in mental health care, as we found that only 28.3% of adults who tested positive for depression and/or anxiety had contact with any provider, largely due to barriers such as financial constraint, busy schedule, self‐reliant attitude, preference for traditional healing, and lack of awareness on available services. These results warrant efforts to close the gap in coverage for care through addressing barriers.

## Graphical abstract

A substantial mental health contact coverage gap (71.7%) exists in rural Nepal, with fewer than one‐third of individuals with depression or anxiety accessing formal care. Financial barriers, self‐reliance, alternative care preferences, and limited awareness were the leading hurdles to treatment seeking.

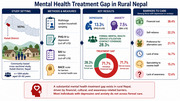

## 1 Introduction

Mental diseases especially depression and anxiety are prevalent and have considerable negative impacts, particularly in developing nations (Kessler et al. 2009). Evidence indicate common mental illness accounts for 1 in 6 years of global impairment (Mental Disorders 2025; Arias et al. 2022). In 2019, depressive disorders ranked 13th while anxiety ranked eighth among the top 25 causes of disability‐adjusted life year (DALY). The economic burden is immense, estimated at 5 trillion USD (Arias et al. 2022). Despite the availability of effective interventions, common mental disorders like depression and anxiety continue to exhibit a substantial contact coverage gap, which could be narrowed in resource‐limited contexts through community‐delivered care models and the redistribution of mental health tasks among trained nonspecialist providers (mhGAP 2025; van Ginneken et al. 2013).

Globally, there is a pronounced gap between demand and actual mental health service utilization, with earlier estimates suggesting that nearly 90% of individuals affected with depression or anxiety do not receive the formal care they need (Santomauro et al. 2024). Furthermore, overall 80% of the mentally ill people do not have access even to the minimal standards of treatment (Moitra et al. 2022).

This burden, coupled with the gap in care, is especially pronounced in low‐ and middle‐income countries (LMICs), where only about 5%–8% of individuals with mental illness receive the necessary care, in contrast to 30%–50% in high‐income countries (Santomauro et al. 2024; Moitra et al. 2022). Based on previous studies conducted in the central region of Nepal, there are 77%–91% of people with common mental disorders who often receive inadequate or no treatment at all (Luitel et al. 2017; National Mental Health Survey Nepal 2020). Mental illness is contributing to 18% of Nepal's noncommunicable disease burden making it one of the serious public health concerns in recent years (World Health Organization, 2014).

Untreated mental conditions pose a significant threat to global public health (Prince et al. 2007), hindering progress toward Sustainable Development Goal Target 3.4, which aims to reduce premature mortality from noncommunicable diseases, including mental health conditions. In particular, untreated depression and anxiety are interrelated and contribute to a range of adverse outcomes, such as discordant relationships with family and peers (WHO 2020), diminished work performance (Luitel et al. 2020; Reardon et al. 2017), reduced productivity, poor quality of life (Luitel et al. 2020), and premature mortality (Cuijpers and Smit 2002). These undesirable consequences underscore the urgent need for early detection and timely management of depression and anxiety, alongside efforts to close the mental health contact coverage gap by identifying and addressing these barriers (Khanal, Selvamani, and Sapkota 2025).

In recent years, multiple global initiatives, including those implemented in Nepal, have sought to mitigate these adverse effects by decreasing the contact coverage gap for common mental illness (Luitel et al. 2015). Nepal has made strides by developing policies and protocols and prioritizing Mental Health Gap Action Programme (mhGAP) implementation at the primary care level (Nepal WHO Special Initiative for Mental Health Situational Assessment 2021). However, the gap in care is substantial and meaningful progress has yet to be achieved (Luitel et al. 2015; Nepal WHO Special Initiative for Mental Health Situational Assessment 2021), especially in the backward provinces of Nepal including Sudurpashchim province where there is a prevailing lack of insufficient evidence to direct the implementation of effective strategies (mhGAP 2025; Luitel et al. 2015).

To date, funding and resources dedicated to mental care in Nepal and other developing nations remain inadequate, despite evidence identifying these countries as epicenters of the global burden of mental illness (mhGAP 2025; Luitel et al. 2015). Handful of evidence from Nepal have highlighted multiple reasons for the existent contact coverage gap such as stigma (WHO 2020; Khanal, Selvamani, and Sapkota 2025; Khanal et al. 2023), misconceptions (Khanal, Selvamani, and Sapkota 2025; Luitel et al. 2015; Khanal, Selvamani, Ghimire, et al. 2025), limited mental health literacy (Khanal, Selvamani, and Sapkota 2025; Luitel et al. 2015; Khanal, Selvamani, Ghimire, et al. 2025), prolonged waiting time (Khanal, Selvamani, and Sapkota 2025), shortages of qualified human resources (WHO 2020; Khanal, Selvamani, and Sapkota 2025; Khanal, Selvamani, Ghimire, et al. 2025), financial limitations (WHO 2020; Khanal, Selvamani, and Sapkota 2025), high treatment expenses (Khanal, Selvamani, and Sapkota 2025; Khanal et al. 2023), and mistrust between users and care providers (Khanal, Selvamani, Ghimire, et al. 2025).

Although mental health is increasingly recognized as a public health priority in Nepal (Luitel et al. 2017; Nepal WHO Special Initiative for Mental Health Situational Assessment 2021), evidence from western and underdeveloped districts remain limited, where the likelihood of a higher contact coverage gap is greater as indicated by earlier research. Kailali, a far‐western district in the Sudurpaschim Province of Nepal, is socioculturally diverse and resource‐constrained making it an important yet understudied setting to explore the utilization and challenges of mental health service (Luitel et al. 2017; Khanal, Selvamani, and Sapkota 2025). Hence, to bridge this gap in the literature, the present study aims to assess the contact coverage gap, and to understand the barriers contributing to it. The objectives of this research are to document the coverage gap among adults with depression and anxiety and to explore possible barriers in mental health care.

## 2 Methodology

### 2.1 Study Area, Design, and Participants

The study employed a community‐based cross‐sectional design among adults aged 20–60 years who had been residing for a minimum of 6 months in the selected site. The study area was Kailali district of Sudurpashchim, Nepal. Sudurpashchim is a backward province in terms of health and development, located at the far‐western end of the country from Nepal's capital city (District Coordination Committee Kailali, 2025). Kailali district is located in Nepal's far‐western province and has a population of 775,709. The literacy rate of Kailali district is 66%, which is lower than the national average. To ensure transparency and comprehensive reporting, this study adhered to the Strengthening the Reporting of Observational Studies in Epidemiology (STROBE) guidelines. The completed STROBE checklist is provided in File S1.

### 2.2 Sample Size and Sampling

The sample size was calculated using Cochran's formula for estimating population proportions. Sample size calculation *n* = *z* ^2^ *pq*/*d* ^2^, *p* = 0.16, *q* = 0.84, *d* = precision of the study = 0.03 (3%) adding 2% design effects and 15% nonresponse rate. Based on this, the calculated sample size was approximately 1319, which was rounded to 1320 for convenience.

Three staged sampling techniques were chosen to select sampling unit. In the first phase, three *Nagarpalikas* (Dhangadhi, Godawari, and Gauriganga) and three *Gaupalikas* (Kailari, Joshipur, and Bardagoriya) were selected by lottery method. In the second phase, wards within the selected *Nagarpalikas* and *Gaupalikas* were chosen. Wards were considered as the primary sampling units (PSUs), and a probability proportionate to size technique was applied to identify the wards.

In the third (final) phase, a list of total households present in the selected wards was obtained from the respective ward office. Based on this, we calculated the **k‐th** interval for household selection. From each selected household, one adult respondent was chosen.


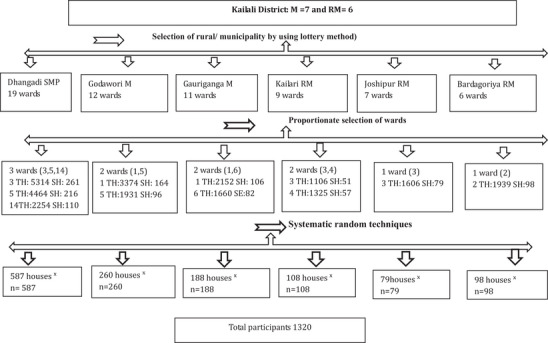


*Note*: ˣ denotes one adult from each house through lottery; assumption: Each household contains at least one adult (20–60 years).

Abbreviations: M, municipality; RM, rural municipality; SH, selected household; SMP, sub‐metropolitan; TH, total household; TP, total population.

### 2.3 Measurement

A door‐to‐door interview schedule was performed by using standardized and validated tools for depression (Patient Health Questionnaire [PHQ‐9]), anxiety (Beck Anxiety Inventory [BAI]), and barriers (Barriers to Access to Care Evaluation [BACE]) instruments.

### 2.4 PHQ‐9

The PHQ‐9 serves as a broadly recognized and extensively validated tool for systematically screening individuals to detect the presence and severity of depressive symptoms. This self‐reported screening tool, PHQ‐9, has been widely used in various medical settings and validated for community settings. It consists of nine questions and responses were recorded on a 0–3 scale, where 0 indicates “not at all” and 3 indicates “always.” Each participant was evaluated to the extent in which they had experienced nine frequently reported depressive symptoms over the preceding 2 weeks (Gilbody et al. 2007). The PHQ‐9 has undergone translation and psychometric validation for use within the Nepali context. A threshold score of 10 or above demonstrates a sensitivity of 94% and a specificity of 80% for identifying a current episode of moderate‐to‐severe depression. Individuals who attained a PHQ‐9 score of 10 or higher were classified as meeting the criteria for depressive disorder (Kohrt et al. 2016; Lund and Garman 2020).

### 2.5 BAI

The BAI is a validated, widely used tool for measuring the level of anxiety of various groups of people in various settings. The instrument was initially developed in 1988 and subsequently underwent a revision in 1993, which incorporated modifications in scoring methodology. It consists of a 21 item self‐reported scale having four‐point Likert‐type scale scored from 0 to 3 with 0 representing “not at all” and 3 denoting “severe” form. Scores on the BAI are categorized into four levels of anxiety severity: minimal (0–7), mild (8–15), moderate (16–25), and severe (30–63) (de Beurs et al., 1997) The BAI has been translated and validated in the context of Nepal. BAI demonstrated strong internal reliability, evidenced by a Cronbach's alpha coefficient of 0.91, sensitivity of 70.9%, and specificity of 73.1% (Adhikari 2019). Those with the score of 16 and above were considered as screening positive for anxiety disorders.

### 2.6 BACE

The BACE scale is a self‐reported validated tool to measure stigma and non‐stigma–related barriers for mental health care. The BACE is 30‐item, four‐point Likert scale ranging from 0 (not at all) to 3 (a lot), which retrieved information from the participants about barriers that ever stopped, delayed, or discouraged them from receiving or continuing care for their mental health problems (Clement et al. 2012). Results for each barrier can be reported in three forms: (1) the mean score for the item, (2) the proportion of respondents identifying it as a barrier to any degree (i.e., selecting 1, 2, or 3), and (3) the proportion identifying it as a major barrier (i.e., selecting 3) (Van Ommeren et al. 1999). Internal consistency of the Nepali version of BACE is Cronbach's alpha 0.91 (Van Ommeren et al. 1999).

### 2.7 Need for Care

In this study, need for care was operationalized based on screened positive for depression and/or anxiety using the PHQ‐9 ≥ 10 or BAI ≥ 16 scale. Individuals’ score above the established cut‐off were considered to have probable depression and anxiety and therefore potential need for mental health care, although this does not represent a formal clinical diagnosis.

### 2.8 Contact Coverage Measurement for Depression or Anxiety

The adults screened positive for depression (PHQ‐9 ≥ 10) and/or anxiety (BAI ≥ 16) were asked whether they had sought for any form of care within the past 12 months. The data collection application (Kobo tool software) automatically prompted follow‐up questions for those exceeding these thresholds. Providers were categorized as specialist mental health professionals, general health practitioners, or traditional/spiritual healers. Contact coverage, as used by (Luitel et al. 2017), refers to the proportion of screened‐positive individuals who had visited any of these providers for care within the past year. The contact coverage measurement is based on self‐reported consultation with any care provider including traditional/spiritual healers for depressive and/or anxiety symptoms. Nevertheless, this measurement of contact coverage doesn't necessary reflect the attainment of evidence‐based mental health care.

### 2.9 Contact Coverage Gap Measurement for Depression or Anxiety

The term “contact coverage gap” in this study is the proportion of adults with elevated depressive or anxiety symptoms (PHQ‐9 ≥ 10 and/or BAI ≥ 16) who self‐reported that they did not access any form of care (specialist mental health professionals, general health practitioners, or traditional/spiritual healers) in the past 12 months. This technique measures contact coverage gap rather than receiving evidence‐based mental health care gap. Furthermore, this operational definition likely differs from the true proportion of individuals with a clinical need for minimally adequate treatment but provides a pragmatic indicator of unmet needs in these settings.

### 2.10 Sociodemographic and Health Measures

The sociodemographic and health measures included were age, sex, caste, religion, marital status, occupation, education, current history of mental illness in the family, past history of mental illness in the family, previous exposure to mental health awareness program, perceived family support, and morbidity status.

### 2.11 Data Collection Techniques

Two research assistants fluent in Nepali and local (Tharu and Doteli) language holding undergraduate medical degrees were selected after conducting an interview. They underwent 15 days of training, which included interview techniques especially for PHQ, BAI, and BACE questionnaire, building rapport, obtaining informed consent, applying inclusion and exclusion criteria, understanding the questionnaire content, and participating in the field test. Data were collected by using ODK kit (Kobo tool software). Data cleaning was done by using Microsoft Excel. For data analysis IBM Statistical Package for the Social Sciences (SPSS) version 20 was used.

### 2.12 Analysis Procedure

Out of 1320 samples, 16 were excluded due to missing data on the primary outcome measure. This study presents the distribution of sociodemographic variables within the sample, the proportion of individuals meeting diagnostic thresholds for depression and anxiety disorders, and the corresponding rates of service utilization across mental health specialists and other categories of care providers. Additionally, we examined the relationship between contact coverage gap and BACE subscale and their sociodemographic characteristics through regression analysis.

## 3 Results

Table 1 summarizes sample characteristics and prevalence of depression and anxiety.

**TABLE 1:** Sociodemographic characteristics of participants and distribution of depression and anxiety (*n* = 1304).

Variables	Total sample % (*n* = 1304)	Screened positive for depression (*n*% = 173/13.3)	Screened positive for anxiety (*n*% = 98/7.5)
**Age (years)**			
21–35	44 (574)	12.4 (71)	8 (46)
36–50	43.5 (567)	14.5 (82)	7.8 (44)
50 and above	12.5 (163)	12.3 (20)	4.9 (8)
**Sex**			
Male	33.7 (439)	8.9 (39)	5.5 (24)
Female	66.3 (865)	15.5 (134)	8.6 (74)
**Caste**			
Brahmin/Chhetri	40.9 (533)	14.4 (77)	10.1 (54)
Others	59.1 (771)	12.5 (96)	5.7 (44)
**Religion**			
Christianity	3.8 (50)	26 (13)	16 (8)
Others (Hindu, Muslim, and Buddhist)	96.2 (1254)	12.8 (160)	7.2 (90)
**Marital status**			
Married	93.3 (1217)	12.9 (157)	7.4 (90)
Unmarried	4.9 (64)	14.1 (9)	10.9 (7)
Others	1.8 (23)	30.4 (7)	4.3 (1)
**Occupation**			
Agriculture	12 (157)	10.8 (17)	2.5 (4)
Business	17.6 (229)	7.9 (18)	5.2 (12)
Service	10.6 (138)	13.8 (19)	9.4 (13)
Foreign employment	1.8 (24)	25 (6)	12.5 (3)
Student	3.1 (40)	12.5 (5)	17.5 (7)
Housewife	46.3 (604)	16.1 (97)	9.1 (55)
Others	8.6 (112)	9.8 (11)	3.6 (4)
**Education**			
Never been to school	32 (417)	16.8 (70)	7.4 (31)
School education	49.6 (47)	11.6 (75)	7.1 (46)
Above school education	18.4 (240)	11.7 (28)	8.8 (21)
**Current history of mental illness in the family**			
No	93.3 (1271)	11.6 (141)	6.3 (77)
Yes	6.7 (87)	36.8 (32)	24.1 (21)
**Past history of mental illness in the family**			
No	95.2 (1241)	12.8 (159)	7.6 (94)
Yes	4.8 (63)	22.2 (14)	6.3 (4)
**Previous exposure to mental health awareness program**			
No	98.9 (1290)	13.1 (169)	7.5 (97)
Yes	1.1 (14)	28.6 (4)	7.1 (1)
**Perceived family support**			
Supportive	95.2 (1241)	11.9 (148)	7.2 (89)
Not supportive	4.8 (63)	39.7 (25)	14.3 (9)
**Morbidity**			
No	81.1 (1058)	94.2 (163)	92.9 (91)
Yes	18.9 (246)	5.8 (10)	7.1 (7)

Most of the respondents were aged 21–50 years (87.5%), females (63.3%), and married (93.3%). About 40.9% belonged to Brahmin/Chhetri caste, and 32% had no formal schooling. Nearly half were housewives (46.3%), followed by business owners (17.6%) and agricultural workers (12%). Few had reported a family history of mental illness (current 6.7%, past 4.8%), exposure to mental health awareness (1.1%), or lack of family support/access to care (5%).

Of the total 1304 participants, 13.3% screened positive for depression and 7.5% for anxiety. Depression was more prevalent among females (15.5%), Christians (26%), separated/divorced/widowed (30.8%), foreign employers (25%), those without family support (39.7%), and those with family history of mental illness (current 36.8%, past 22.2%). Anxiety was higher among females (8.6%), Brahmin/Chhetris (10.1%), unmarried (10.9%), students (17.5%), foreign employees, those with positive current family history (24.1%), and those without family support (14.3%).

Table 2 highlights the percentage of the participants who had contact with provider from a mental health specialist, general practitioner, or traditional providers for symptoms related to depression and anxiety. Overall, 28.3% of the participants who were screened positive for depression or/and anxiety reported contact with service providers, while 30.1% with depression and 34.7% with anxiety patients stated that they had contact with providers during the preceding year.

**TABLE 2:** Contact coverage among adults with depression and/or anxiety (*n* = 198).

Factors	Total % (*n*) 15.2 (198)	Depression % (*n*) 13.3 (173)	Anxiety % (*n*) 7.5 (98)
**Contact with any provider within the past year**				
No	71.7 (142)	69.9 (121)	65.3 (64)
Yes	28.3 (56)	30.1 (52)	34.7 (34)
Type of service providers*multiple response *n* = 56				
Specialist mental health providers	92.9 (52)	87.5 (49)	57.1 (32)
Generalist health providers	62.5 (35)	53.6 (30)	50 (28)
Traditional providers	98.2 (55)	71.4 (40)	89.3 (50)

Among the participants who visited service providers, 98.2%, 62.5%, and 92.9% consulted with traditional providers, generalist health providers, and specialist mental health providers, respectively. These findings indicated that traditional healers were the most popular source of care, often in combination with other biomedical providers.

**FIGURE 1:**
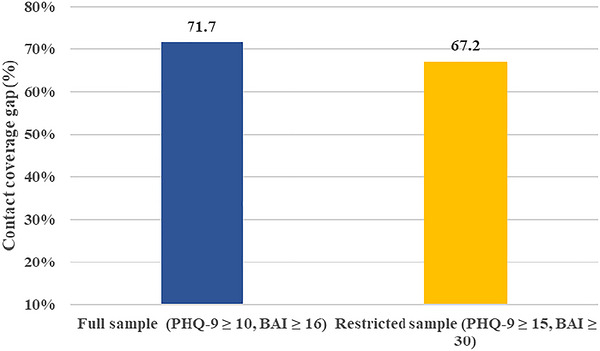
Contact coverage among adults with depression and/or anxiety according to degree of severity (*n* = 198).

Figure 1 illustrates the contact coverage gap among adults with depression and/or anxiety, comparing the full sample and participants with higher symptom severity (PHQ‐9 ≥ 15 and/or BAI ≥ 30). The contact coverage gap remained substantial even with individuals restricted to higher score for severity.

Table 3 illustrates the perceived barriers to care, encompassing both “any degree of barrier” and “major barrier,” alongside the average scores for every BACE statement among individuals diagnosed with depression and anxiety.

**TABLE 3:** Barriers to accessing mental health services among individuals with depression and/or anxiety who have not received care from any provider.

Barriers to mental health care	Item mean	Barriers to any degree % (*n*)	Major barriers % (*n*)
**Stigma‐related barriers**			
“Concern that I might be seen as weak for having a mental health problem”	0.59	51	5.6
“Concerns that might harm my chances when applying for jobs”	1.50	17.7	3
“Concern about what my family think, say, do or feel”	0.35	32.3	2.5
“Feeling embarrassed or ashamed”	0.34	31.3	2
“Concern that I might be seen as crazy”	0.29	26.3	3
“Concern that I might be seen as a bad parent”	0.87	20.2	2.5
“Concern that people I know might find out”	0.19	18.7	0.5
“Concern that people might not take me seriously if they found out I was having mental health care”	0.19	17.7	1
“Not wanting a mental health problem to be on my medical records”	0.04	4	0
“Concern that my children may be taken into care or that I may lose access or custody without my agreement”	0.8	14.1	1.5
“Concern about what my friends might think, say or do”	0.18	15.2	2
“Concern about what people at work might think, say or do”	1.10	11.1	2
**Financial barriers**			
“Problems with transport or travelling to appointments”	0.45	42.4	3
“Not being able to afford the financial costs involved”	1.33	74.2	38.4
“Difficulty taking time off work”	2.01	50	16.7
**Cultural beliefs and practices**			
“Preferring to get alternative forms of care (e.g., traditional/religious healing or alternative/complementary therapies”	0.75	48	21.7
“Professionals from my own ethnic or cultural group not being available”	0.07	6.6	0
“Preferring to get help from family or friends”	0.06	5.1	0.5
“Fear of being put in hospital against my will”	0.25	24.2	0.5
**Low perceived needs**			
“Wanting to solve the problems on my own”	1.07	80.3	22.2
“Thinking the problem would get better by itself”	0.88	71.7	15.2
“Dislike of talking about my feelings, emotions or thoughts”	0.22	22.2	0
“Thinking I did not have a problem”	0.18	17.2	1
**Lack of knowledge about the available care**			
“Being unsure where to go to get mental health care”	0.69	53.5	12.6
**Perceived ineffectiveness of available services**			
“Concerns about the treatments available (e.g., medication side effects)”	0.19	19.2	1
“Thinking that mental health care probably would not help”	0.26	24.2	1.5
“Having had previous bad experiences with mental health staff”	0.06	5.6	0.5
**Lack of Support**			
“Having problems with childcare while I receive mental health care”	0.81	12.6	3
“Being too unwell to ask for help”	0.21	18.7	2
“Having no one who could help me get mental health care”	0.19	15.7	2.5

For depression and anxiety, every statement evaluated for experiencing “any degree of barriers” ranged from 4% (item—“Not wanting a mental health problem to be on my medical records”) to 80.3% (item—“Wanting to solve the problems on my own”). The most commonly indicated barriers were preferring to get it solved on their own (80.3%), unaffordable financial cost (74.4%), speculating it would get better with time (71.7%), confusion over service places for mental health care (53.5%), concern about seeming weak (51%), and busy schedule (50%).

The proportion of the respondents indicating these barriers as a “major barrier” ranged from 0% to 34.4%. The most commonly indicated major barriers were unaffordable cost (38.4%); self‐solving attitude (22.2%); preference for alternative types of care, for example, traditional or religious healing (21.7%); busy schedule (16.7%); speculating it would resolve by itself (15.2%); unsureness about where to seek help (12.6%); and concern with being reflected as weak related to a mental health problem (5.6%)

Associated factors with contact coverage of the people with anxiety and depression were measured utilizing unadjusted logistic regression, as presented in Table 4. Results showed that presence of morbidity among screened positive respondents for depression and anxiety has higher odds of care‐seeking attitude. While the overall BACE score was not associated with care‐seeking nature, participants with lower score on lack of awareness about available services subscale of BACE had lower odds of receiving mental health care (OR = 0.60, CI = 0.38, 0.95; *p* = 0.029).

**TABLE 4:** Factors associated with contact coverage from health care providers in the past 12 months.

Factors	Contact coverage		Unadjusted odds ratio (95% CI)		*p* value
	Yes *n* (%)	No *n* (%)			
**Sex**					
Male	15 (31.9)	32 (68.1)	1		
Female	41 (27.2)	110 (72.8)	1.26 (0.62, 2.56)		0.527
**Religion**					
Other	51 (28)	131 (72)	1		
Christianity	5 (31.3)	11 (68.8)	0.78 (0.28, 2.59)		0.78
**Marital status**					
Married	53 (29.8)	125 (70.2)	1		
Unmarried	2 (15.4)	11 (84.6)	2.33 (0.50, 10.9)		0.28
Others	1 (14.3)	6 (85.7)	2.54 (0.29, 21.7)		
**Education**					
No formal school education	22 (28.6)	55 (71.4)	1		
Formal school education	34 (28.1)	87 (71.9)	1.02 (0.54, 1.93)		0.94
**Occupation**					
Agriculture	5 (29.4)	12 (70.6)	1		
Business	9 (45)	11 (55)	0.51 (0.13, 1.99)		0.33
Service	9 (40.9)	13 (59.1)	0.60 (0.16, 2.31)		0.46
Foreign employment	2 (33.3)	4 (66.7)	0.85 (0.11, 6.11)		0.86
Student	1 (10)	9 (90)	3.75 (0.37, 37.9)		0.26
Housewife	25 (23.3)	87 (77.7)	1.45 (0.47, 4.51)		0.52
Others	5 (45.5)	6 (54.5)	0.50 (0.10, 2.43)		0.39
**Morbidity status**					
No	29 (21.2)	108 (78.8)	1		
Yes	27 (44.3)	34 (55.7)	2.96 (1.54, 5.67)		0.001
	**No** **(mean ± SD)**	**Yes** **(mean ± SD)**	**Odds ratio (95% CI)**	***p* value**	
**Barriers to mental health care**					
Overall, BACE	10.80 (7.10)	9.58 (7.08)	0.97 (0.93, 1.02)	0.28	
Stigma	2.95 (3.44)	2.34 (3.21)	0.94 (0.85, 1.04)	0.25	
Financial barriers	2.45 (1.53)	2.46 (1.58)	1.08 (0.93, 1.29)	0.09	
Low perceived needs	2.22 (1.42)	2.13 (1.34)	0.96 (0.77, 1.18)	0.68	
Cultural practice and beliefs	1.29 (1.38)	1.29 (1.37)	0.99 (0.79,1.25)	0.96	
Lack of support	0.59 (1.07)	0.41 (0.82)	0.82 (0.58, 1.16)	0.27	
Lack of knowledge about available services	0.76 (0.72)	0.50 (0.65)	0.60 (0.38, 0.95)	0.029	
Perceived ineffectiveness of services	0.53 (0.80)	0.46 (0.72)	0.91 (0.63, 1.32)	0.64	

## 4 Discussion

This is to our knowledge the first community‐based population study in Sudurpashchim province of Nepal to explore contact coverage gap and the specific barriers in receiving mental health services among individuals screened positive for depression or anxiety.

The magnitude of depression in this study (13.3%) is remarkably similar to the pooled estimate of depression in 30 countries estimated from 68 studies (12.9%) (Lim et al. 2018). The magnitude of depression in the present study was higher than that found in studies conducted in Chitwan district of Nepal (11.5%) (Luitel et al. 2017), the National Mental Health Survey report (2.9%) (National Mental Health Survey Nepal 2020), and a study in Uttarakhand, India (6%) (Mathias et al. 2015). Meanwhile, a study conducted based on the National Health Demographic Survey (2022), showed that depression (4%) was lower and anxiety (17.7%) was higher than the present study (Pandey et al. 2024). Magnitude of the mental illness varied across provinces, ranging highest in Koshi province (13.9%) and lowest in Madhesh province (2.1%) of Nepal (National Mental Health Survey Nepal 2020). The variation in depression and anxiety across different provinces and districts in Nepal can be caused by a number of factors including limited accessibility to mental health care, cultural beliefs, socioeconomic disparities, and environmental vulnerabilities (National Mental Health Survey Nepal 2020; Thornicroft et al. 2017).

Nearly one‐third of the individuals screened positive for depression and anxiety had sought for any form of care from various care providers in the past 12 months. This indicated that considerable disparities existed among mental health services. This denotes a significant contact coverage gap, underscoring the necessity to counteract the obstacles encountered by these patients. This figure significantly exceeded the disparity observed in other developing nations, where more than half (52.6%) of individuals with depression received mental health care in the preceding 12 months (Thornicroft et al. 2017). Two recent systematic reviews done in global scale also highlighted that the mental health service coverage for depression was extremely low ranging from 5% to 8% (Santomauro et al. 2024; Moitra et al. 2022). The magnitude of the contact coverage gap identified in the present study was comparatively lower than that documented in previous studies conducted in China (3.4%) (Shen et al. 2006), Korea (1.9%) (Cho et al. 2009), and Northern India (100%) (Mathias et al. 2015). However, compared to studies conducted in high income countries like the United States (65.7%) (Kohn et al. 2018), the United Kingdom (53.4%) (NHS England 2025), and Canada (63.4%) (Mental Disorders in Canada 2022), the present study observed a higher gap in contact coverage among the respondents. Nonetheless, a global review showed consistent results highlighting that there is 72% gap in care for anxiety disorder (Alonso et al. 2018). The high proportion of participants reporting contact with traditional healers in this study also highlighted the important role of nonmedical providers in mental health seeking context. Moreover, the definition of contact coverage was deliberately broad and included traditional and spiritual healers in the present study, reflecting real‐world scenario of help‐seeking patterns. However, this approach means that we cannot determine the proportion of participants who received minimally adequate, evidence‐based mental health care, which may contribute to persistent unmet needs. Therefore, the true gap in access to such care may be even larger than the 71.7% contact coverage gap reported in this study.

The National Mental Health Survey (2020) report of Nepal reported a contact coverage gap of 77% (National Mental Health Survey Nepal 2020), while a community‐based study conducted in Chitwan district by Luitel et al. (2017) documented an even higher coverage gap of 91%, compared to the present study (71.7%), which is still substantial. Nevertheless, this reflected a gradual reduction trend from 91% in 2017 to 77% in 2020 which suggests a positive trend in service utilization, potentially reflecting increased mental health awareness as a result of expansion of government and nongovernmental service programs, and inclusion of mental health into primary healthcare system in certain districts (Luitel et al. 2017; Khanal et al. 2023). In spite of all these efforts, the persistently higher contact coverage gap highlighted that a large proportion of individuals still remain untouched and untreated. Evidence suggest that complex, interlinked, multilevel challenges exists while accessing MHS for patients in SAARC nations which are contributing to a higher level of gap in mental health service utilization (Khanal, Selvamani, Ghimire, et al. 2025).

Those challenges are at individual, organizational, societal, and structural (policy) levels at various phases of the care pathway. Evidence highlighted that depression and anxiety at primary care is largely unrecognized (Culpepper 2003) and structural and health system weakness, including scarce mental health and human services (Demyttenaere et al. 2004) as well as lack of awareness along with cost of care (Khanal, Selvamani, and Sapkota 2025) and stigma perceived by the people with mental illness further limit the treatment accessibilities which are in support of the current findings (Khanal, Selvamani, and Sapkota 2025; Khanal, Selvamani, Ghimire, et al. 2025). In line with the study findings, a qualitative study has highlighted that patients with depression and anxiety commonly access traditional healers initially (Luitel et al. 2025). Factors such as support of the family or friend or community and service quality and mental health awareness influence care‐seeking behavior.

A considerable number of participants who did not seek care over the past 12 months reported facing majority of the barriers. One in every four respondents reported that they had experienced these as “major barriers.” The commonly stated major barriers (> 12%) were limited financial resources, willingness to solve problem by self, preferring alternative treatments, difficulty sparing time from work, thinking that the problem would self‐resolve, and being unsure where to receive mental health care. This revealed the presence of significant barriers which massively hindered the seeking habit of depressive and anxious patients leading to a significant contact coverage gap. Studies from Korea (Cho et al. 2009), Nepal (Luitel et al. 2017; National Mental Health Survey Nepal 2020), Pakistan (Atif et al. 2016), and the United Kingdom (Smith et al. 2019) also showed consistent results for not seeking care which were financial hardship, wanting to handle the problem on their own, having no insight of the disease, and belief that it would resolve on its own. Moreover, reliable evidence considered (Kessler et al. 2009) unawareness and stigma, (Mental Disorders 2025) limited resources (human and material), and (Arias et al. 2022) limited dissemination to be the major challenges while closing the contact coverage gap in low‐income countries. Studies from 46 Sub‐Saharan African (Komu et al. 2025) and 8 SAARC (Khanal, Selvamani, Ghimire, et al. 2025) countries observed lack of awareness on mental disorders, and available professional services, adverse attitudes toward mental health care and professionals, cultural and religious beliefs leading to overdependence on traditional care, stigmatizing beliefs, and discrimination from family/community/healthcare providers were the contributing factors behind the contact coverage gap, further consolidating the findings of the current study.

Contact coverage was positively associated with the presence of morbidity. One plausible explanation is that individuals already diagnosed with a health issue may have more frequent visits and adequate contact with healthcare providers, thereby increasing opportunities for referral, recognition of symptoms, or early initiation of treatment. Previous studies also have similarly reported that comorbidity with chronic physical illnesses often lead to higher mental health service utilization, partly due to increased interaction with the healthcare system and heightened perceived need for care (Jimenez et al. 2015). Likewise, contact coverage was negatively associated with lack of knowledge on existing mental health services. This aligns with evidence from developing countries, and also from underserved regions in high‐income countries, showing that informational barriers often outweigh other factors in determining the service use (Khanal, Selvamani, and Sapkota 2025; Khanal, Selvamani, Ghimire, et al. 2025).

### 4.1 Policy Implications

The outcomes of this study have implications for development of comprehensive, multilevel strategies by revising the existing policies and addressing the persistent contact coverage gap despite a decade's initiative for depression and anxiety in Kailali district of Sudurpaschim province. Although the gradual decrease in the disparity over the past few years is encouraging, a significant number of individuals affected remain untreated as a result of financial constraints, a lack of awareness about available services, reliance on traditional healers, and work‐related barriers. Hence, this study will inform and encourage policymakers to prioritize the integration of traditional healers into mental health services, reinforcement of referral systems, and assurance of the availability of essential pharmaceuticals and trained personnel at the local level by fostering collaboration between biomedical and traditional care providers, instituting financial protection mechanisms to reduce out‐of‐pocket costs, and expanding community‐based awareness programs to improve mental health literacy. Furthermore, targeted outreach in marginalized districts, in conjunction with workplace and community‐level interventions, are indispensable for enhancing the cultural acceptability, equity, and accessibility of mental health care.

### 4.2 Strength and Limitations

This the first kind of study conducted in Kailali district of Sudurpashchim province by utilizing the validated Nepali version tool to measure depression, anxiety, and barriers to access care which is the key strength of the present study. Second, we employed random sampling technique which enhanced the possibilities of better representation of study area and population.

However, the major limitation was the inclusion of a significantly large percentage of the female sample (66.3%) in the current study whereas the percentage of the female group of Kailali district was 52.1% only (District Coordination Committee Kailali, 2025) which was due to a trend of male labor migration to India. Hence, generalizability of the results may be limited among those who were not present in the district during survey. This study has an additional limitation related to the assessment of need for care. The operational definition used may differ from the true proportion of individuals with a clinical need for minimally adequate treatment/care, although it serves as a pragmatic indicator of unmet needs in these settings. Likewise, this study relied on PHQ‐9 and BAI as screening instruments rather than diagnostic interviews. While both tools have good sensitivity and specificity in the Nepali context, a score above the chosen cut‐offs does not necessarily imply a clinical diagnosis or an immediate need for formal treatment. Our estimates may therefore overstate the number of individuals who would meet full diagnostic criteria. However, the fact that only 28.3% of screened‐positive adults had contact with any provider, and that substantial barriers were reported across multiple domains, indicate that the under‐provision of care remains large even under more conservative assumptions about “true” need. Additionally, we did not explicitly stratify contact coverage on the basis of symptoms severity. Although sensitivity analyses using different cut‐off scores for PHQ‐9 and BAI were conducted, a more detailed assessment across severity categories could provide better insight into whether service utilization varied with the degree of depressive or anxiety symptoms. Another limitation of this study is that the broad definition of contact coverage included traditional and spiritual healers. While this reflects a real‐world help‐seeking pattern, it limits the ability to determine whether participants received a minimally adequate, evidence‐based mental health care.

## 5 Conclusion

The contact coverage gap for depression and anxiety is substantial in Kailali district due to a range of barriers such as financial constraints, busy schedules, self‐reliant attitudes, preference for traditional healers, and lack of awareness about available services. Such a pattern is understandable in Nepal, given that out‐of‐pocket payments represent over 50% of total healthcare expenditure (World Bank Group n.d.) and prevailing misconceptions and stigma is enormously high due to unawareness and cultural beliefs. Hence, to reduce the substantial contact coverage gap, strategies to enhance mental health literacy are essential, along with further research to identify additional barriers that can guide the development of targeted interventions.

## Author Contributions

**Gayatri Khanal**: conceptualization, investigation, writing – original draft, methodology, writing – review and editing, formal analysis, project administration, software, resources, data curation. **Y. Selvamani**: conceptualization, investigation, supervision, formal analysis, writing – review and editing, methodology, software. **Prabhat Sapkota**: conceptualization, writing – review and editing, methodology, project administration, supervision, resources, validation, data curation.

## Funding

The authors have nothing to report.

## Ethics Statement

Ethical clearance for this study was obtained from SRM Institute of Science and Technology (SRMIST), Institutional Review Board (IRB) (00109/IEC/2024), and Ethical Review Board (ERB) of Nepal Health Research Council (NHRC) (340, 2024). All of the study's procedure was conducted in accordance with the Declaration of Helsinki.  All the municipalities and rural municipalities were officially contacted with letters, and permission was obtained. Similarly, official approval was taken from selected wards of various municipalities and rural municipalities, hospitals, and schools. The study was thoroughly explained to each and every participant, and written consent was obtained from each individual. When required, consent was also obtained from guardians. Those who were screened positive for depression and anxiety and had no medical visit were suggested to visit the nearest health center with appropriate communication.

## Conflicts of Interest

The authors declare no conflicts of interest.

## Supporting information




**Supporting Material**: STROBE Statement—Checklist of items included in reports of observational studies

## Data Availability

Data will be available from the corresponding author upon reasonable request.

## References

[brb371551-bib-0005] Adhikari, C. 2019. “Application and Validation of the Beck Anxiety Inventory Among Nepalese School Adolescents.” Journal of Health and Allied Sciences 9, no. 1: 51–58.

[brb371551-bib-0006] Alonso, J. , Z. Liu , S. Evans‐Lacko , et al. 2018. “Treatment Gap for Anxiety Disorders Is Global: Results of the World Mental Health Surveys in 21 Countries.” Depression and Anxiety 35, no. 3: 195–208. https://pubmed.ncbi.nlm.nih.gov/29356216/. 29356216 10.1002/da.22711 PMC6008788

[brb371551-bib-0007] Arias, D. , S. Saxena , and S. Verguet . 2022. “Quantifying the Global Burden of Mental Disorders and Their Economic Value.” eClinicalMedicine 54: 101675. https://pmc.ncbi.nlm.nih.gov/articles/PMC9526145/. 36193171 10.1016/j.eclinm.2022.101675 PMC9526145

[brb371551-bib-0008] Atif, N. , K. Lovell , N. Husain , S. Sikander , V. Patel , and A. Rahman . 2016. “Barefoot Therapists: Barriers and Facilitators to Delivering Maternal Mental Health Care Through Peer Volunteers in Pakistan: A Qualitative Study.” International Journal of Mental Health Systems 10, no. 1: 24. https://pmc.ncbi.nlm.nih.gov/articles/PMC4793537/. 26985235 10.1186/s13033-016-0055-9 PMC4793537

[brb371551-bib-0009] de Beurs, E. , K. A. Wilson , D. L. Chambless , A. J. Goldstein , and U. Feske . 1997. “Convergent and divergent validity of the Beck Anxiety Inventory for patients with panic disorder and agoraphobia.” Depression and Anxiety 6, no. 4: 140–146. 10.1002/(SICI)1520-6394(1997)6:4<140::AID-DA2>3.0.CO;2-G. https://www.researchgate.net/publication/292088125_Beck_anxiety_inventory. 9559283

[brb371551-bib-0010] Cho, S. J. , J. Y. Lee , J. P. Hong , H. B. Lee , M. J. Cho , and B. J. Hahm . 2009. “Mental Health Service Use in a Nationwide Sample of Korean Adults.” Social Psychiatry and Psychiatric Epidemiology 44, no. 11: 943–951. https://link.springer.com/article/10.1007/s00127‐009‐0015‐7. 19294325 10.1007/s00127-009-0015-7

[brb371551-bib-0011] Clement, S. , E. Brohan , D. Jeffery , C. Henderson , S. L. Hatch , and G. Thornicroft . 2012. “Development and Psychometric Properties the Barriers to Access to Care Evaluation Scale (BACE) Related to People With Mental Ill Health.” BMC Psychiatry 12, no. 1: 1–11. https://bmcpsychiatry.biomedcentral.com/articles/10.1186/1471‐244X‐12‐36. 22546012 10.1186/1471-244X-12-36 PMC3379935

[brb371551-bib-0012] Cuijpers, P. , and F. Smit . 2002. “Excess Mortality in Depression: A Meta‐Analysis of Community Studies.” Journal of Affective Disorders 72, no. 3: 227–236. https://pubmed.ncbi.nlm.nih.gov/12450639/. 12450639 10.1016/s0165-0327(01)00413-x

[brb371551-bib-0013] Culpepper, L. 2003. “Use of Algorithms to Treat Anxiety in Primary Care.” Journal of Clinical Psychiatry 64, no. Suppl 2: 30–33. https://pubmed.ncbi.nlm.nih.gov/12625797/. 12625797

[brb371551-bib-0014] Demyttenaere, K. , R. Bruffaerts , J. Posada‐Villa , et al. 2004. “Prevalence, Severity, and Unmet Need for Treatment of Mental Disorders in the World Health Organization World Mental Health Surveys.” JAMA 291, no. 21: 2581–2590. https://pubmed.ncbi.nlm.nih.gov/15173149/. 15173149 10.1001/jama.291.21.2581

[brb371551-bib-0046] District Coordination Committee, Kailali (Nepal): Government of Nepal (n.d.) . 2025. https://dcckailali.gov.np/.

[brb371551-bib-0015] Gilbody, S. , D. Richards , S. Brealey , and C. Hewitt . 2007. “Screening for Depression in Medical Settings With the Patient Health Questionnaire (PHQ): A Diagnostic Meta‐Analysis.” Journal of General Internal Medicine 22, no. 11: 1596–1602. https://pubmed.ncbi.nlm.nih.gov/17874169/. 17874169 10.1007/s11606-007-0333-y PMC2219806

[brb371551-bib-0042] van Ginneken, N. , P. Tharyan , S. Lewin , et al. 2013. “Non‐Specialist Health Worker Interventions for the Care of Mental, Neurological and Substance‐Abuse Disorders in Low‐ and Middle‐Income Countries.” Cochrane Database of Systematic Reviews 2013, no. 11: CD009149. https://www.cochranelibrary.com/cdsr/doi/10.1002/14651858.CD009149.pub2/full. 10.1002/14651858.CD009149.pub2 24249541

[brb371551-bib-0016] Jimenez, D. E. , B. L. Cook , G. Kim , et al. 2015. “Relationship Between General Illness and Mental Health Service Use and Expenditures Among Racially‐Ethnically Diverse Adults ≥ 65 Years.” Psychiatric Services 66, no. 7: 727–733. https://pubmed.ncbi.nlm.nih.gov/25772763/. 25772763 10.1176/appi.ps.201400246 PMC4490047

[brb371551-bib-0017] Kessler, R. C. , S. Aguilar‐Gaxiola , J. Alonso , et al. 2009. “The Global Burden of Mental Disorders: An Update From the WHO World Mental Health (WMH) Surveys.” Epidemiologia e Psichiatria Sociale 18, no. 1: 23–33. https://pubmed.ncbi.nlm.nih.gov/19378696/. 19378696 10.1017/s1121189x00001421 PMC3039289

[brb371551-bib-0018] Khanal, G. , Y. Selvamani , S. Ghimire , S. Thapa , and R. Dhital . 2025. “Examining Barriers to Access Mental Health Services Among Patients With Mental Health Issues in SAARC Nations: A Systematic Review and Meta‐Synthesis of Qualitative Studies.” Asian Journal of Psychiatry 103: 104331. https://www.sciencedirect.com/science/article/abs/pii/S1876201824004246. 39631131 10.1016/j.ajp.2024.104331

[brb371551-bib-0019] Khanal, G. , Y. Selvamani , and P. Sapkota . 2023. “Insights on Historical Milestones of Mental Health in Nepal: Country Profile.” Indian Journal of Psychiatry 65, no. 11: 1122–1128. https://pmc.ncbi.nlm.nih.gov/articles/PMC10795665/. 38249153 10.4103/indianjpsychiatry.indianjpsychiatry_197_23 PMC10795665

[brb371551-bib-0020] Khanal, G. , Y. Selvamani , and P. Sapkota . 2025. “Exploring Barriers and Facilitators of Mental Health Care in Sudurpaschim Province, Nepal: A Socioecological Qualitative Study of Patients With Depression and Anxiety and Health Care Professionals.” BMC Health Services Research 25, no. 1: 1–11. https://bmchealthservres.biomedcentral.com/articles/10.1186/s12913‐025‐12983‐4. 40597294 10.1186/s12913-025-12983-4 PMC12219968

[brb371551-bib-0022] Kohn, R. , A. A. Ali , V. Puac‐Polanco , et al. 2018. “Mental Health in the Americas: An Overview of the Treatment Gap.” Revista Panamericana de Salud Pública 42: e165. https://pmc.ncbi.nlm.nih.gov/articles/PMC6386160/. 31093193 10.26633/RPSP.2018.165 PMC6386160

[brb371551-bib-0023] Kohrt, B. A. , N. P. Luitel , P. Acharya , and M. J. D. Jordans . 2016. “Detection of Depression in Low Resource Settings: Validation of the Patient Health Questionnaire (PHQ‐9) and Cultural Concepts of Distress in Nepal.” BMC Psychiatry 16, no. 1: 1–14. https://bmcpsychiatry.biomedcentral.com/articles/10.1186/s12888‐016‐0768‐y. 26951403 10.1186/s12888-016-0768-y PMC4782581

[brb371551-bib-0024] Komu, C. K. , M. Ngigi , and A. J. Melson . 2025. “Barriers and Facilitators to Accessing Mental Health Services for Adults in Sub‐Saharan Africa: A Systematic Review.” Mental Health Science 3, no. 1: e70006. 10.1002/mhs2.70006.

[brb371551-bib-0025] Lim, G. Y. , W. W. Tam , Y. Lu , C. S. Ho , M. W. Zhang , and R. C. Ho . 2018. “Prevalence of Depression in the Community From 30 Countries Between 1994 and 2014 /692/699/476/1414 /692/499 Article.” Scientific Reports 8, no. 1: 1–10. https://www.nature.com/articles/s41598‐018‐21243‐x. 29434331 10.1038/s41598-018-21243-x PMC5809481

[brb371551-bib-0026] Luitel, N. P. , M. J. Jordans , A. Adhikari , et al. 2015. “Mental Health Care in Nepal: Current Situation and Challenges for Development of a District Mental Health Care Plan.” Conflict and Health 9, no. 1: 3. https://pmc.ncbi.nlm.nih.gov/articles/PMC4331482/. 25694792 10.1186/s13031-014-0030-5 PMC4331482

[brb371551-bib-0027] Luitel, N. P. , M. J. D. Jordans , B. A. Kohrt , S. D. Rathod , and I. H. Komproe . 2017. “Treatment Gap and Barriers for Mental Health Care: A Cross‐Sectional Community Survey in Nepal.” PLoS ONE 12, no. 8: e0183223. 10.1371/journal.pone.0183223. 28817734 PMC5560728

[brb371551-bib-0028] Luitel, N. P. , M. J. D. Jordans , P. Subba , and I. H. Komproe . 2020. “Perception of Service Users and Their Caregivers on Primary Care‐Based Mental Health Services: A Qualitative Study in Nepal.” BMC Family Practice 21, no. 1: 1–11. https://bmcprimcare.biomedcentral.com/articles/10.1186/s12875‐020‐01266‐y. 32988367 10.1186/s12875-020-01266-y PMC7523041

[brb371551-bib-0029] Luitel, N. P. , B. Lamichhane , K. Sah , et al. 2025. “Facilitators in Treatment Pathways for Depression or Anxiety Among Adults in Nepal: A Qualitative Study.” BMC Public Health 25, no. 1: 1–13. https://bmcpublichealth.biomedcentral.com/articles/10.1186/s12889‐025‐23225‐x. 40457314 10.1186/s12889-025-23225-x PMC12128395

[brb371551-bib-0030] Lund, C. , and A. E. Garman . 2020. “Treatment Coverage, Barriers to Care and Factors Associated With Help‐Seeking Behaviour of Adults With Depression and Alcohol Use Disorder in Chitwan District, Nepal.” Master's thesis, University of Cape Town. http://hdl.handle.net/11427/32404.

[brb371551-bib-0031] Mathias, K. , I. Goicolea , M. Kermode , L. Singh , R. Shidhaye , and M. S. Sebastian . 2015. “Cross‐Sectional Study of Depression and Help‐Seeking in Uttarakhand, North India.” BMJ Open 5, no. 11: e008992. https://pubmed.ncbi.nlm.nih.gov/26589428/. 10.1136/bmjopen-2015-008992 PMC4663438 26589428

[brb371551-bib-0003] Mental Disorders in Canada . 2022. Government of Canada. https://www150.statcan.gc.ca/n1/pub/11‐627‐m/11‐627‐m2023053‐eng.htm?utm_source=chatgpt.com.

[brb371551-bib-0004] Mental Disorders . 2025. WHO. https://www.who.int/news‐room/fact‐sheets/detail/mental‐disorders.

[brb371551-bib-0032] mhGAP . Mental Health Gap Action Program: Scaling Up Care for Mental, Neurological and Substance Use Disorders . 2025. WHO. https://iris.who.int/handle/10665/43809. 26290926

[brb371551-bib-0033] Moitra, M. , D. Santomauro , P. Y. Collins , et al. 2022. “The Global Gap in Treatment Coverage for Major Depressive Disorder in 84 Countries From 2000–2019: A Systematic Review and Bayesian Meta‐Regression Analysis.” PLOS Medicine 19, no. 2: e1003901. 10.1371/journal.pmed.1003901. 35167593 PMC8846511

[brb371551-bib-0001] National Mental Health Survey Nepal . 2020. Government of Nepal Nepal Health Research Council. https://nhrc.gov.np/publication/report‐of‐national‐mental‐health‐survey‐2020/.

[brb371551-bib-0002] Nepal WHO Special Initiative for Mental Health Situational Assessment . 2021. WHO. https://www.who.int/publications/m/item/nepal‐‐‐who‐special‐initiative‐for‐mental‐health.

[brb371551-bib-0034] NHS England . 2025. “Chapter 2: Mental Health Treatment and Service Use.” NHS England. Published November 27, 2025. https://digital.nhs.uk/data‐and‐information/publications/statistical/adult‐psychiatric‐morbidity‐survey/survey‐of‐mental‐health‐and‐wellbeing‐england‐2023‐24/mental‐health‐treatment‐and‐service‐use.

[brb371551-bib-0035] Pandey, A. R. , B. Adhikari , B. Bista , et al. 2024. “Prevalence, Determinants and Care‐Seeking Behaviour for Anxiety and Depression in Nepalese Population: A Secondary Analysis of Data From Nepal Demographic and Health Survey 2022.” BMJ Open 14, no. 8: e078582. https://bmjopen.bmj.com/content/14/8/e078582. 10.1136/bmjopen-2023-078582 PMC11308907 39107021

[brb371551-bib-0036] Prince, M. , V. Patel , S. Saxena , et al. 2007. “No Health Without Mental Health.” Lancet 370, no. 9590: 859–877. https://pubmed.ncbi.nlm.nih.gov/17804063/. 17804063 10.1016/S0140-6736(07)61238-0

[brb371551-bib-0037] Reardon, T. , K. Harvey , M. Baranowska , D. O'Brien , L. Smith , and C. Creswell . 2017. “What Do Parents Perceive Are the Barriers and Facilitators to Accessing Psychological Treatment for Mental Health Problems in Children and Adolescents? A Systematic Review of Qualitative and Quantitative Studies.” European Child & Adolescent Psychiatry 26, no. 6: 623–647. https://pubmed.ncbi.nlm.nih.gov/28054223/. 28054223 10.1007/s00787-016-0930-6 PMC5446558

[brb371551-bib-0038] Santomauro, D. F. , T. Vos , H. A. Whiteford , D. Chisholm , S. Saxena , and A. J. Ferrari . 2024. “Service Coverage for Major Depressive Disorder: Estimated Rates of Minimally Adequate Treatment for 204 Countries and Territories in 2021.” Lancet Psychiatry 11, no. 12: 1012–1021. https://www.thelancet.com/action/showFullText?pii=S2215036624003171. 39572105 10.1016/S2215-0366(24)00317-1 PMC11579305

[brb371551-bib-0039] Shen, Y.‐C. , M.‐Y. Zhang , Y.‐Q. Huang , et al. 2006. “Twelve‐Month Prevalence, Severity, and Unmet Need for Treatment of Mental Disorders in Metropolitan China.” Psychological Medicine 36, no. 2: 257–267. https://pubmed.ncbi.nlm.nih.gov/16332281/. 16332281 10.1017/S0033291705006367

[brb371551-bib-0040] Smith, M. S. , V. Lawrence , E. Sadler , and A. Easter . 2019. “Barriers to Accessing Mental Health Services for Women With Perinatal Mental Illness: Systematic Review and Meta‐Synthesis of Qualitative Studies in the UK.” BMJ Open 9, no. 1: e024803. https://pubmed.ncbi.nlm.nih.gov/30679296/. 10.1136/bmjopen-2018-024803 PMC6347898 30679296

[brb371551-bib-0041] Thornicroft, G. , S. Chatterji , S. Evans‐Lacko , et al. 2017. “Undertreatment of People With Major Depressive Disorder in 21 Countries.” British Journal of Psychiatry 210, no. 2: 119–124. https://pubmed.ncbi.nlm.nih.gov/27908899/. 10.1192/bjp.bp.116.188078 PMC5288082 27908899

[brb371551-bib-0043] Van Ommeren, M. , B. Sharma , S. Thapa , et al. 1999. “Preparing Instruments for Transcultural Research: Use of the Translation Monitoring Form With Nepali‐Speaking Bhutanese Refugees.” Transcultural Psychiatry 36, no. 3: 285–301.

[brb371551-bib-0044] WHO . Mental Health Atlas . 2020. WHO. https://www.who.int/publications/i/item/9789240036703.

[brb371551-bib-0045] World Bank Group . n.d. “Glossary | DataBank.” Accessed August 12, 2025. https://databank.worldbank.org/metadataglossary/africa‐development‐indicators/series/SH.XPD.OOPC.TO.ZS.

[brb371551-bib-0021] World Health Organization . 2014. “Multisectoral action plan for the prevention and control of noncommunicable diseases (2014–2020): Nepal.” https://www.who.int/docs/default-source/nepal-documents/multisectoral-action-plan-for-prevention-and-control-of-ncds-(2014-2020).pdf?sfvrsn=c3fa147c_4.

